# Unveiling hepatitis E virus diversity in Sudan’s internally displaced populations: a molecular epidemiology approach

**DOI:** 10.1186/s41182-025-00864-9

**Published:** 2025-12-12

**Authors:** Hytham A. Osman, Wafaa M. K. Bakr, Mona H. Hashish, Isam M. Elkhidir, Shahinaz Bedri, Samuel O. Oyola, Helene Norder, Ekram W. Abd El-Wahab

**Affiliations:** 1 Department of Microbiology, High Institute of Public Health, Alexandria University, Alexandria, 21561 Egypt; 2 Epidemiology Department, National Public Health Laboratory, Khartoum, Sudan; 3 Department of Microbiology and Parasitology, Faculty of Medicine, University of Khartoum, Khartoum, Sudan; 4 National Public Health Laboratory, Khartoum, Sudan; 5 Department of Pathology and Laboratory Medicine, Ahfad University for Women, Khartoum, Sudan; 6 International Livestock Research Institute (ILRI), Nairobi, Kenya; 7 Sahlgrenska University Hospital, 10000–20000 Stockholm County, Gothenburg, Sweden; 8 Department of Tropical Health, High Institute of Public Health, Alexandria University, 165 El Horreya Road, Alexandria, 21561 Egypt

**Keywords:** Hepatitis E virus, HEV-1e, Genotyping, Phylogenetic analysis, Internally displaced persons, Sudan

## Abstract

**Background:**

Hepatitis E virus (HEV) is recognized as a cause of acute viral hepatitis, particularly in low-resource and humanitarian settings, although its burden varies across different populations and geographic areas. Internally displaced persons (IDPs) are at high risk due to inadequate sanitation and contaminated water. This study aimed to characterize the molecular epidemiology and genotypes of HEV among IDPs in Al-Azaza Camp, Blue Nile State, Sudan.

**Methods:**

A cross-sectional study was conducted from August to December 2021 during the rainy season. Serum samples from 1,078 participants were screened for anti-HEV IgM and IgG antibodies. A subset of 20 IgM-positive samples was selected for molecular analysis using real-time RT-PCR. Eighteen high-quality RNA-positive samples were sequenced, and genotyping was performed based on the ORF2 region. Phylogenetic analysis was conducted using the HEV Genotyping Tool and Geneious Prime software.

**Results:**

Overall, 75.6% of participants tested positive for IgG antibodies. All sequenced isolates (*n* = 18) were classified as genotype 1, subtype 1e (HEV-1e), closely related to *Paslahepevirus balayani*. The isolates clustered with reference strains from Chad and Nigeria, indicating regional circulation and genetic conservation of HEV-1e in sub-Saharan Africa.

**Conclusion:**

Despite the single-site scope and low RNA yield limitations, the study findings align with regional HEV-1e circulation patterns and emphasize the need for sustained surveillance and consideration of cross-border transmission.

**Supplementary Information:**

The online version contains supplementary material available at 10.1186/s41182-025-00864-9.

## Introduction

Hepatitis E virus (HEV) is a globally significant, enterically transmitted pathogen responsible for an estimated 20 million infections and over 40,000 deaths annually, predominantly in low- and middle-income countries [1]. As a cause of acute viral hepatitis, HEV poses a disproportionate threat in settings characterized by inadequate sanitation, contaminated water supplies, and high population mobility. These conditions are especially prevalent in conflict-affected regions such as Sudan, where decades of armed conflict, political instability, and environmental crises have led to widespread displacement of populations. Internally displaced persons (IDPs) in Sudan often reside in overcrowded camps with limited access to safe water and healthcare, making them particularly vulnerable to waterborne diseases such as hepatitis E [2, 3].

HEV is a small, non-enveloped or quasi-enveloped virus with a single-stranded, positive-sense RNA genome of approximately 7.2 kilobases [4]. It belongs to the *Hepeviridae* family, genus *Orthohepevirus A*, and comprises at least eight genotypes (HEV-1 to HEV-8), four of which (HEV-1 to HEV-4) are known to infect humans [5, 6]. HEV-1 and HEV-2 are strictly human pathogens associated with large-scale waterborne outbreaks in regions with limited sanitation infrastructure. In contrast, HEV-3 and HEV-4 are zoonotic and responsible for sporadic, foodborne infections in industrialized nations, often linked to the consumption of undercooked pork and wild game [5, 6]. HEV-3, in particular, exhibits considerable genetic diversity and a wide host range [7].

Despite increasing global recognition of HEV’s public health impact, data on its genetic diversity and transmission dynamics in sub-Saharan Africa, including Sudan, remain sparse. Most available studies have focused on outbreak reports and seroprevalence surveys, with limited molecular characterization of circulating strains. This knowledge gap is particularly concerning in IDP settings, where the convergence of environmental risk factors and high population turnover may promote the emergence and spread of diverse HEV variants.

Furthermore, recent findings suggest that zoonotic transmission may also contribute to the HEV burden in Africa. Animal reservoirs such as pigs, goats, and cattle have been shown to harbor HEV-like viruses in several African countries, including Egypt, Uganda, and the Democratic Republic of the Congo [8, 9].. In Sudan, HEV antibodies have been detected in donkeys, although the specific genotypes involved have not been determined [2]. These findings underscore the need for comprehensive molecular surveillance to evaluate the potential role of zoonotic transmission and the diversity of HEV strains circulating among both human and animal populations.

Molecular epidemiology offers a critical framework for addressing these gaps by combining genomic sequencing, phylogenetic analysis, and field epidemiology to uncover the evolutionary relationships and transmission patterns of viral pathogens [10, 11]. By applying this approach in IDP communities in Sudan, this study aims to provide a deeper understanding of HEV genotype distribution, genetic diversity, and possible zoonotic linkages in one of the world’s most under-investigated HEV-endemic regions.

This investigation represents one of the first efforts to characterize HEV at the molecular level in Sudan’s displaced populations. The findings will offer vital insights into the virus' evolutionary dynamics, help identify circulating strains with epidemic potential, and support the development of targeted public health interventions to mitigate HEV transmission in humanitarian settings.

## Methods

### Study design, setting, and population

A cross-sectional study was conducted from August to December 2021, during the rainy season, a period associated with heightened HEV transmission in humanitarian settings [12]. The study was conducted in Al-Azaza Camp, Al-Roseires district, Blue Nile State, Sudan. Established in 2015, the camp houses over 4,000 IDPs living in temporary shelters with limited access to clean water and sanitation. Blue Nile State, bordering Ethiopia and South Sudan, comprises six districts spanning 45,844 km^2^ and has a predominantly rural, ethnically diverse population of approximately 1.2 million.

### Sample size and sampling method

The required sample size of 1,078 participants was determined using PASS software (version 19.0.1), based on a prior HEV seroprevalence study among IDPs in South Sudan [3], which reported an IgG seroprevalence of 71% and 4% IgM positivity. Calculations applied a 5% alpha error and 1% precision. Stratified random sampling was used based on age and sex distributions from a comparable IDP population in Darfur. A preliminary survey of Al-Azaza Camp generated a household list; households were randomly selected using computer-generated numbers and a fixed interval method until the target sample size was reached. A total of 1,078 IDPs aged one year and above, including males, females, and pregnant women, were enrolled in the study.

### Sample collection and processing

Venous blood (3 mL) was collected aseptically from each participant using vacutainer tubes. After clotting and centrifugation, serum was separated, aliquoted, and stored at − 80 °C for further testing.

### Serological testing

All serum samples were screened for anti-HEV IgM and IgG using commercial ELISA kits (Fortress Diagnostics, UK), following the manufacturer’s instructions.

### Molecular characterization of HEV infecting genotypes

#### Viral RNA extraction and cDNA synthesis

Viral RNA was extracted from 140 µl serum using the QIAamp^®^ Viral RNA Mini Kit (QIAGEN, Cat. No. 52906, Germany). Briefly, samples were lysed under denaturing conditions to inactivate RNases and release intact viral RNA. After adjusting buffer conditions for optimal RNA binding, lysates were loaded onto QIAamp mini spin columns. RNA adhered to the membrane, contaminants were washed away, and RNA was eluted in RNase-free buffer, yielding high-purity RNA suitable for immediate use or storage. Buffer AVE was added to lyophilized carrier RNA (1 µg/µl). The RNA was aliquoted and stored at − 30 to − 15 °C. Prior to mixing with buffer AVL, the buffer was checked for precipitates and incubated at 80 °C if necessary. Volumes were calculated per sample batch as follows:

$${\text{n }} \times \, 0.{\text{56 ml }} = {\text{ y ml,}}$$

$${\text{y ml }} \times { 1}0 \, \mu {\text{l}}/{\text{ml }} = {\text{ z }}\mu {\text{l,}}$$

where *n* = sample number, *y* = buffer AVL volume, *z* = carrier RNA-AVE volume.

Gentle mixing of AVL was done by inversion. Buffer AW1 was prepared by adding 130 ml ethanol to 98 ml AW1 concentrate; AW2 was prepared with 160 ml ethanol to 66 ml concentrate.

#### Viral RNA purification (spin protocol)

560 µl of AVL-carrier RNA was combined with 140 µl serum, mixed, and incubated for 10 min at room temperature. Post-centrifugation, 560 µl ethanol was added, mixed, and the sample was loaded onto the QIAamp column. The process included sequential washes with 500 µl buffers AW1 and AW2. Final elution was done with 60 µl buffer AVE into a 1.5-ml tube, yielding ≥ 90% recovery.

#### Real-time PCR

Real-time RT-PCR was performed using the RealStar^®^ HEV RT-PCR Kit (Altona Diagnostic, Germany), designed for use with the available instrument, Rotor-Gene^®^ Q5/6 plex Platform (QIAGEN), according to the manufacturer’s procedure. The Kit Components contained all essential PCR reagents including HEV-specific primers/probes and internal control (IC). Four quantification standards were used for positive controls or standard curve generation. RT-PCR combined reverse transcription, target amplification, and fluorescence-based detection using FAM^™^-labeled HEV probes and JOE-labeled IC probes in a one-tube format. The steps included cDNA synthesis, PCR amplification, and amplicon detection. Reagents were thawed, mixed, and briefly centrifuged. The heterologous IC served as an RT-PCR inhibition control. Twenty-five µl of master mix was added to each well/tube, followed by 25 µl of sample or control. Reactions were mixed, sealed, centrifuged (1000 × g, 30 s), and run on the thermalcycler per software instructions.

### Data analysis

Valid runs required detection of positive control (FAM^+^ and JOE^+^) and negative control (FAM^−^ and JOE^+^). Invalid runs lacked these criteria.

Result interpretation positive: FAM^+^ and JOE^+^. Negative: FAM^−^ and JOE^+^. Inhibited: FAM^−^ and JOE^−^. Repeats are required if only FAM^−^ and JOE^+^ were detected.

### Sequencing and genotyping

*Sequencing*: Sanger sequencing detection and quantification of HEV RNA was undertaken as follows: all 20 seropositive and RNA-positive HEV samples were sequenced within a 1.3 kb area of HEV ORF2. cDNA was synthesized using the SuperScript II^®^ Reverse Transcriptase kit (Thermo Fisher Scientific™) according to the manufacturer’s protocol with 10 μM of the reverse primer R3O (5′-AGACTCCCGGGTTTTACCTACCTTCATTTT-3′). In a semi-nested PCR, 5 μl of cDNA was performed using MyFi Mix^®^ (Bioline, London, UK) reagents. In the first round of PCR, primers R3O and ORF2-FWD1 (5′-TTGGCGTGACCAGKCCCAGCGCC-3′) were used, and in the second round, primers R3O and ORF2-G3 (5′-TCYAAYTAYGCYCAGTAYCGGGT-3′) were used. For both rounds, the cycle conditions for amplification were the same: initial denaturation at 92 °C for 2 min, followed by 35 cycles of (denaturation at 95 °C for 15 s, annealing at 52 °C for 30 s, and elongation at 72 °C for 2 min) then a final elongation stage of 10 min at 72 °C for 10 min. The PCR products were revealed on a 1.5% agarose gels and purified using Illustra GFX^®^ PCR DNA and GEL BAND^®^ Purification Kit (GE Healthcare, Illinois, and USA). The purified PCR products were quantified and then sequenced on an ABI 3730^®^ automated capillary sequencer (Thermo Fisher Scientific™) using the primers R3O, ORF2-FWD1, ORF2-G3, MENG-F1 (5′-GTYATGYTYTGCATACATGGCT-3′), MENG-R1-FWD (5′-GACAGAATTGATTTCGTCGGC-3′), and MENG-R1 (5′-AGCCGACGAAATYAATTGT GTC-3′), and MENG-R0 (5′ − CCCTTATCCTGCTGAGCATTCTC-3′).

### Phylogenetic analysis

Phylogenetic analysis was conducted to determine the genotypes of the HEV isolates identified in this study. A total of 20 HEV-positive samples were subjected to genotyping based on the nucleotide sequences obtained from the amplified ORF2 region following sequence editing. Sequence alignment and preliminary genotype assignment were carried out using the Hepatitis E Virus Genotyping Tool (Version 1.10), which enables automated classification based on sequence similarity to known genotype references. To validate these results and assess sequence homology, each sequence was further analyzed using the Basic Local Alignment Search Tool (BLAST) provided by the National Center for Biotechnology Information (NCBI) database (https://www.ncbi.nlm.nih.gov/). The edited sequences were aligned against HEV reference sequences to determine the closest genetic relatives. Subsequently, a phylogenetic tree was constructed using Geneious Prime software, which allowed for visualization of the evolutionary relationships and confirmation of genotype assignment through clustering with reference strains.

## Results

### Serological results

Among 1,078 participants, anti-HEV IgM was detected in 20 (1.86%) individuals, while IgG seropositivity was observed in 815 (75.6%). Of the 20 IgM-positive samples, those with sufficient RNA quality were sequenced, resulting in 18 high-quality HEV sequences suitable for genotyping.

### Molecular analysis of HEV-RNA

Molecular detection of HEV RNA was performed using real-time reverse transcription RT-PCR on a subset of 20 serum samples that demonstrated strong serological reactivity to HEV-specific IgM. Of these, 9 samples were positive for HEV IgM alone, while 11 samples tested positive for both HEV IgM and IgG antibodies. These samples were selected from the total cohort of 1,078 participants based on their serological profiles. The RT-PCR analysis was employed to confirm the presence of active viral replication and to enable subsequent molecular characterization of the circulating HEV strains (Table 1).

**Table 1:** Detection of HEV in serum samples of IDPs by RT-qPCR

Samples	*n*	Detection of HEV-RNA by RT-qPCR
		*n* (%)
Positive HEV IgM	9	9 (45.0)
Positive HEV IgM/IgG	11	11 (55.0)
Total	20	20 (100.0)

Of the 20 IgM-positive serum samples selected for molecular analysis, all (100.0%) tested positive for HEV RNA by real-time RT-PCR (Table S2). This unusually high PCR positivity rate is atypical compared to commonly reported detection rates and likely reflects the specific criteria used for sample selection. These 20 samples were chosen from a larger IgG-positive cohort based on IgM titers and early symptom onset, which may have enriched the subset for cases with active viral replication and higher viral loads. This selection process likely contributed to the elevated RNA detection rate observed in this study. A flowchart provides an overview of inclusion/exclusion criteria at each point (Figure S1).

### HEV genotyping and phylogenetic analysis

**Fig. 1:**
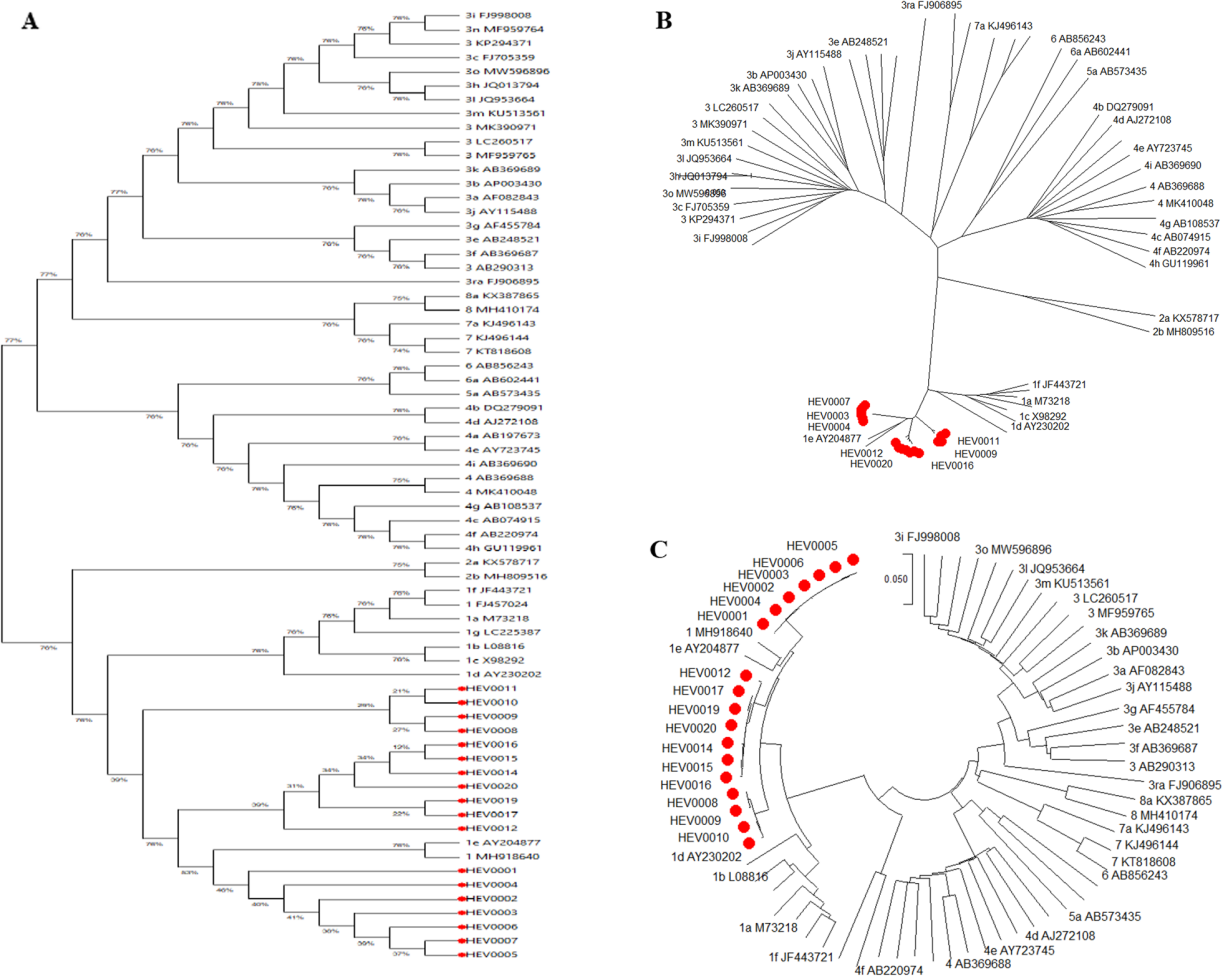
Evolution analysis—phylogenetic tree within Orthohepevirus A subtypes, based on neighbor-joining method. Study stains in red color. A; classic tree, B; radiation tree, C; circle tree

Based on sequencing data, 18 out of the 20 HEV RNA-positive samples yielded high-quality sequences suitable for phylogenetic analysis. All 18 HEV-1e isolates have been submitted to GenBank under accession numbers SAMN52637457 to SAMN52637474, associated with BioProject PRJNA1344343 (Table S1 for metadata table). Genogroup classification of these isolates revealed that all were closely related to *Paslahepevirus balayani*, the causative agent of human hepatitis E. All isolates were identified as genotype 1 (HEV-1), with further sub-clustering into subtype 1e (HEV-1e), indicating a genetically homogenous group. The genomic sequences obtained from these isolates aligned consistently with the *Orthohepevirus A* (taxon: 1678143), reference genome (NC_001434.1), with defined start and end positions relative to this sequence. \n\nPhylogenetic assignments were initially performed using the HEV Genotyping Tool (version 1.3) and further validated through phylogenetic tree construction using Geneious Prime software (Fig. 1). Phylogenetic tree analyses were performed on two genomic regions of the HEV-positive isolates (designated HEV000x). Further analysis of the 18 high-quality HEV-1e sequences revealed very low intra-subtype genetic diversity among the isolates obtained from the Al-Azaza Camp cohort. The sequences clustered tightly together in the phylogenetic analysis (Fig. 1), with minimal nucleotide differences observed between the individual HEV isolates (e.g., HEV0001, HEV0002, etc.). This high degree of genetic homogeneity suggests the recent circulation of a single, conserved HEV-1e strain within the IDP setting at the time of sampling. The analyses confirmed that one of the isolates clustered with the African clade of genotype 1 subtype e (gt1e), supporting its previous genotypic classification. The number of HEV-positive isolates analyzed in each phylogenetic tree is indicated in parentheses (X). The analysis focused on the highly conserved ORF2 region and involved comparative alignment with representative human HEV reference strains from diverse geographical origins. The GenBank accession numbers of reference strains included in the analysis are shown in Table 2.

**Table 2:** Reference strains of HEV isolates and their geographical location

Isolate	Location
KJ451629, KJ451633, AY 204877	Nigeria
KT833800, KU178916	South Africa
DQ445498, NC_001434	China
KC012634	Cameroon
KP255948-DQ860005, KF95653, KP255925	Canada
AF117275, KP255922	Taiwan
JQ7636-11	France
KJ6459-43	Venezuela
FJ2308-50	India
AF2640-10	Argentina
HM3669-41	Philippines
LC073306, LC022740, AB434138, AB671034, AB671046, LC037975, AB094207, AB073911, AB986280, AB094211, AB476429, AB671052, AB607892, AB094209, AB112743, AB290039, AB298181, AB683185, AB607888, AB607890	Japan
AF336290, AY032756, AF332620, AF336290, AY032758	Netherlands
AF110387, AF110388, AF466681, AF466660, AF020497	USA
AJ344190, AJ428851	United Kingdom

### Evolutionary relationships of 68 taxa

The evolutionary history was inferred using the Neighbor-Joining method [13]. The optimal tree with the sum of branch length = 4.323 is shown. The tree is drawn to scale, with branch lengths in the same units as those of the evolutionary distances used to infer the phylogenetic tree. The evolutionary distances were computed using the Maximum Composite Likelihood method [14] and are in the units of the number of base substitutions per site. The proportion of sites where at least 1 unambiguous base is present in at least 1 sequence for each descendant clade is shown next to each internal node in the tree. The analytical procedure encompassed 68 coding nucleotide sequences using 1st, 2nd, 3rd, and non-coding positions. The pairwise deletion option was applied to all ambiguous positions for each sequence pair resulting in a final data set comprising 9495 positions. Evolutionary analyses were conducted in MEGA12 [15].

## Discussion

This study presents a descriptive analysis of HEV prevalence and molecular characterization in Al-Azaza Camp, a settlement designated for IDPs in Blue Nile State, Sudan. Conducted during the rainy season, when HEV transmission typically peaks due to increased exposure to contaminated water sources. This investigation aligns with prior reports that highlight the seasonal dynamics of HEV outbreaks in similar humanitarian and resource-limited settings [3, 12].

Al-Azaza Camp offers a geographically well-defined and demographically coherent population, which allows for high-resolution spatial and epidemiological analysis. Although the study area is restricted in scale, this limitation is offset by enhanced reliability in sample tracking and population coverage, allowing for accurate estimation of HEV seroprevalence and genotype distribution within the camp.

This detection rate is considerably higher than typically reported in population-based studies, which likely reflects our selective sampling of acutely ill, IgM-positive individuals, thereby enriching for cases with high viral loads.

Phylogenetic classification confirmed that all sequenced isolates belonged to genotype 1 (HEV-1), subtype 1e (HEV-1e), which is part of the Orthohepevirus A species under the recently revised taxonomy *Paslahepevirus balayani* [16]. HEV-1 is considered one of the most conserved genotypes and has been further divided into seven recognized subtypes (1a–1 g), which are typically human-specific and primarily transmitted via the fecal–oral route. The robustness of genetic classification through sequencing has been well-established, and it reflects the ecological and zoonotic epidemiology of HEV strains, thereby supporting the phylogenetic approach as both scientifically sound and epidemiologically relevant.

Due to limitations in RNA yield and sample volume, only 18 of the 20 HEV RNA-positive samples yielded sequences of sufficient quality for phylogenetic analysis. Nonetheless, all successfully sequenced samples clustered within the HEV-1e subtype. This genotype–subtype association is consistent with previous findings from various regions across Africa, where HEV-1e has been repeatedly implicated in both sporadic and large-scale outbreaks. Geographic tracing of HEV-1e reveals its circulation across tropical and subtropical areas, particularly in Africa, with confirmed presence from central regions such as Sudan and Chad [17], to northern countries like Egypt, Tunisia, and Algeria [18, 19], and as far south as Namibia [20]. Additionally, HEV-1e strains have been reported as imported cases in Europe, particularly Spain, likely originating from the Horn of Africa, including Ethiopia [21].

Importantly, HEV-1e has been the predominant subtype associated with major outbreaks in sub-Saharan Africa, highlighting its epidemiological significance and potential for cross-border transmission [22]. The finding that all sequenced isolates belonged to the genetically conserved HEV-1e subtype is further reinforced by the analysis of intra-subtype diversity. The minimal genetic variation observed among the 18 isolates from the camp strongly suggests a single, recent introduction of the virus into the population, most likely driving a common-source outbreak (e.g., contaminated water). This lack of diversity is highly indicative of transmission via a shared contaminated source, such as the limited and often compromised water supply and sanitation systems within the IDP camp setting. Had the infections been due to multiple, disparate introduction events over a long endemic period, a greater degree of genetic variation and potentially multiple subtypes (or diverse strains within HEV-1e) would have been expected. Therefore, the genetic homogeneity observed provides strong molecular epidemiological evidence supporting the hypothesis that the high HEV endemicity in Al-Azaza Camp is primarily driven by an active, single-strain waterborne outbreak cycle, a pattern consistent with HEV-1e epidemiology in sub-Saharan Africa. The findings of the present study align closely with data from Nigeria, where the second complete genome sequence of an HEV-1e strain (NG/17-0503) was reported during a 2017 outbreak. This Nigerian isolate shared a 94.2% nucleotide sequence identity with an HEV-1e strain previously isolated in Chad, underscoring the regional circulation and genetic conservation of this subtype [22]. The finding of exclusively HEV-1e and its close clustering with regional strains (Chad and Nigeria) supports the idea of regional circulation and genetic conservation of this subtype in sub-Saharan Africa. The lack of diversity suggests that, at the time of sampling, the HEV infections in the camp were driven by a single, established HEV-1e strain likely spread through the inadequate water and sanitation conditions prevalent in the IDP setting.

In conclusion, this study offers an epidemiological evidence for high endemicity of HEV among IDP in Sudan. Molecular characterization analysis confirmed that all sequenced isolates belonged to genotype 1, subtype 1e (HEV-1e) within *Paslahepevirus balayani,* aligning with established classifications and the known epidemiology of HEV in the region. Although RNA yield was limited, only 18 of 20 IgM-positive samples produced sequences of sufficient quality, all clustered within HEV-1e, indicating the presence of a genetically conserved strain circulating in the camp at the time of sampling. These findings are consistent with regional and continental data, reinforcing the widespread circulation and genetic stability of HEV-1e in sub-Saharan Africa. While acknowledging limitations in geographic scope and sample yield, this study contributes valuable evidence for ongoing HEV-1e transmission in displacement settings and highlights the importance of continued surveillance, particularly in the context of potential cross-border transmission.

## Supplementary Information

Supplementary Material 1.

## Data Availability

Data are available by the corresponding author upon request.

## Abbreviations

cDNA: Complementary deoxynucleic acid
HEV: Hepatitis E virus
IDPs: Internally displaced persons/people/populations
IgG: Can refer to immunoglobulin G
IgM: Can refer to immunoglobulin M
PCR: Polymerase chain reaction
RNA: Ribonucleic acid
RT: Reverse transcription
