# Supplementary material for: Unveiling hepatitis E virus diversity in Sudan’s internally displaced populations: a molecular epidemiology approach
Source: Trop Med Health. 2025 Dec 12;53:186. doi: 10.1186/s41182-025-00864-9 (PMC12706927; doi:10.1186/s41182-025-00864-9)
Supplement: Supplementary file 1 — Supplementary Material 1. [file 41182_2025_864_MOESM1_ESM.docx]

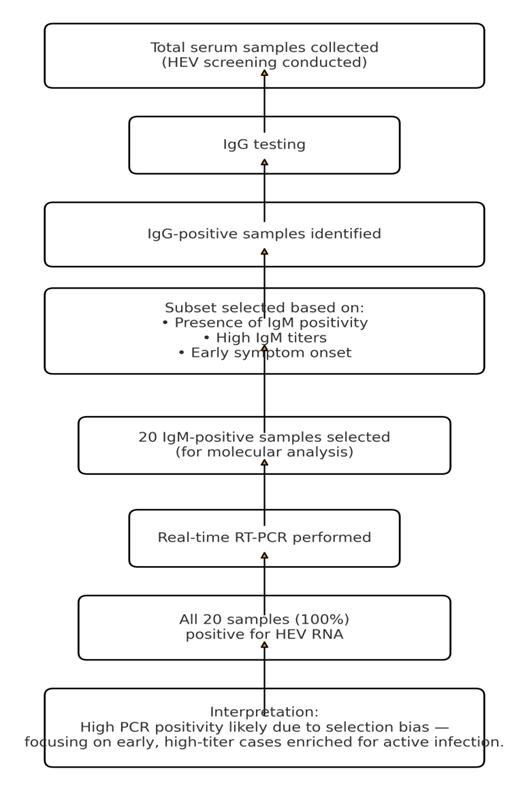


Figure S1: A flowchart providing an overview of the study inclusion/exclusion criteria

| **Accession** | **Sample Name** | **SPUID** | **Organism** | **Tax ID** | **Isolate** | **URL** |
| --- | --- | --- | --- | --- | --- | --- |
| SAMN52637457 | HEV0001 | HEV0001 | Hepatitis E virus | 291484 | Human | https://www.ncbi.nlm.nih.gov/sra/52637457 |
| SAMN52637458 | HEV0002 | HEV0002 | Hepatitis E virus | 291484 | Human | https://www.ncbi.nlm.nih.gov/sra/52637458 |
| SAMN52637459 | HEV0003 | HEV0003 | Hepatitis E virus | 291484 | Human | https://www.ncbi.nlm.nih.gov/sra/52637459 |
| SAMN52637460 | HEV0004 | HEV0004 | Hepatitis E virus | 291484 | Human | https://www.ncbi.nlm.nih.gov/sra/52637460 |
| SAMN52637461 | HEV0005 | HEV0005 | Hepatitis E virus | 291484 | Human | https://www.ncbi.nlm.nih.gov/sra/52637461 |
| SAMN52637462 | HEV0006 | HEV0006 | Hepatitis E virus | 291484 | Human | https://www.ncbi.nlm.nih.gov/sra/52637462 |
| SAMN52637463 | HEV0007 | HEV0007 | Hepatitis E virus | 291484 | Human | https://www.ncbi.nlm.nih.gov/sra/52637463 |
| SAMN52637464 | HEV0008 | HEV0008 | Hepatitis E virus | 291484 | Human | https://www.ncbi.nlm.nih.gov/sra/52637464 |
| SAMN52637465 | HEV0009 | HEV0009 | Hepatitis E virus | 291484 | Human | https://www.ncbi.nlm.nih.gov/sra/52637465 |
| SAMN52637466 | HEV0010 | HEV0010 | Hepatitis E virus | 291484 | Human | https://www.ncbi.nlm.nih.gov/sra/52637466 |
| SAMN52637467 | HEV0011 | HEV0011 | Hepatitis E virus | 291484 | Human | https://www.ncbi.nlm.nih.gov/sra/52637467 |
| SAMN52637468 | HEV0012 | HEV0012 | Hepatitis E virus | 291484 | Human | https://www.ncbi.nlm.nih.gov/sra/52637468 |
| SAMN52637469 | HEV0014 | HEV0014 | Hepatitis E virus | 291484 | Human | https://www.ncbi.nlm.nih.gov/sra/52637469 |
| SAMN52637470 | HEV0015 | HEV0015 | Hepatitis E virus | 291484 | Human | https://www.ncbi.nlm.nih.gov/sra/52637470 |
| SAMN52637471 | HEV0016 | HEV0016 | Hepatitis E virus | 291484 | Human | https://www.ncbi.nlm.nih.gov/sra/52637471 |
| SAMN52637472 | HEV0017 | HEV0017 | Hepatitis E virus | 291484 | Human | https://www.ncbi.nlm.nih.gov/sra/52637472 |
| SAMN52637473 | HEV0019 | HEV0019 | Hepatitis E virus | 291484 | Human | https://www.ncbi.nlm.nih.gov/sra/52637473 |
| SAMN52637474 | HEV0020 | HEV0020 | Hepatitis E virus | 291484 | Human | https://www.ncbi.nlm.nih.gov/sra/52637474 |

Table S1: HEV-1e isolates GenBank with accession numbers and BioProject Metadata

Table S2: Concordance of IgM Serostatus and Viral RNA Detection

**| Serological Status | Total Samples (N) | RNA Positive Samples (n) | RNA Positivity Rate |**

**|--------------------|-------------------|--------------------------|---------------------|**

**| IgM-Positive | 20 | 20 | 100% |**

**| IgM-Negative | [Not specified] | [Not specified] | [Not specified] |**

**| Total | [Not specified] | [Not specified] | [Not specified] |**
